# Plant-Derived Chinese Medicine Monomers on Ovarian Cancer via the Wnt/*β*-Catenin Signaling Pathway: Review of Mechanisms and Prospects

**DOI:** 10.1155/2021/6852867

**Published:** 2021-12-06

**Authors:** Jia-Yue Xu, Fang-Yuan Liu, Shao-Xuan Liu, Liang-Zhen Xie, Jia Li, Yu-Ting Ma, Feng-Juan Han

**Affiliations:** ^1^Department of Obstetrics and Gynecology, Heilongjiang University of Chinese Medicine, Harbin 150040, China; ^2^Department of Obstetrics and Gynecology, The First Affiliated Hospital of Heilongjiang University of Chinese Medicine, Harbin 150040, China

## Abstract

Ovarian cancer (OC) is a common malignant tumor of the female reproductive system and has a high morbidity and mortality rate. The progression and metastasis of OC are complex and involve multiple signaling pathways. The Wnt/*β*-catenin signaling pathway is closely related to OC, and therefore blocking the activation of the Wnt/*β*-catenin signaling directly or inhibiting related genes, and molecular targets is of great value in treating OC. Toxicities such as myelotoxicity, cardiotoxicity, genotoxicity, and vasospasm are the major side effects for common anticancer drugs and are well documented. There is, therefore, a need to develop new, effective, safer, and more affordable anticancer drugs from alternative sources. In recent years, plant-derived Chinese medicine monomers have drawn increasing attention due to their high safety, low toxicity, minimal side effects, and antitumor effects. Plant-derived Chinese medicine monomers are effective against multiple targets and can regulate the growth, proliferation, apoptosis, invasion, and migration of OC as well as reverse drug resistance by regulating the Wnt/*β*-catenin signaling pathway. In this review, we summarize and provide mechanisms and prospects for the use of plant-derived Chinese medicines for the prevention and treatment of OC.

## 1. Introduction

Ovarian cancer (OC) is one of the most common gynecological malignancies and is a serious threat to women's lives and health. OC is insidious, with no typical symptoms in the early stage, and most patients present with stage III/IV disease at the time of diagnosis [1]. There were about 300,000 new cases of OC worldwide in 2018, accounting for 3.4% of the total number of female malignant tumor cases [2]. Ovarian cancer mortality has declined since the mid-1970s due to reductions in incidence and improvements in treatment in recent decades [3]. But despite these advances, the survival rate for OC has changed only modestly in recent decades, even in high-resource countries, such as the United States and Canada, and remains at only 47% five years after diagnosis [4]. The treatment of OC is based chiefly on surgery, adjuvant postoperative chemotherapy or nonadjacent chemotherapy, molecular-targeted therapy, and other comprehensive treatments. About 80% of patients can achieve complete clinical remission through surgery combined with chemotherapy, but there are still patients who cannot accept surgery or the toxic side effects of chemotherapy drugs, which leads to limitations in these methods [5, 6].

Toxicities such as myelotoxicity, cardiotoxicity, genotoxicity, pulmonary toxicity, cutaneous toxicity, and vasospasm are the major side effects for common anticancer drugs, such as 5-fluorouracil, doxorubicin, and bleomycin, and are well documented. There is, therefore, a need to develop new, effective, safer, and more affordable anticancer drugs from alternative sources. Recently, plant-derived Chinese medicines, with a broad target range and low side effects, have begun to play a major role in treating tumors. Many plant-derived Chinese medicines used for treating various tumors have beneficial effects, including inhibiting the occurrence and development of cancer and prolonging the survival time of cancer patients. With the deepening of molecular biology research on the pathogenesis of OC, various signaling pathways that regulate OC have attracted widespread attention, such as the Wnt/*β*-catenin, JAK/STAT, PI3K/AKT, and NF-*κ*B signaling pathways [7, 8]. The use of plant-derived Chinese medicines to target Wnt/*β*-catenin signaling to treat OC has been actively explored by many groups, and the purpose of this review was to summarize this research and to provide mechanisms and prospects for the use of plant-derived Chinese medicines for the prevention and treatment of OC.

## 2. The Wnt/*β*-Catenin Signaling Pathway

As one of the chief signaling pathways in most organisms, the Wnt/*β*-catenin signaling pathway is essential for embryonic development and for adult tissue homeostasis and regeneration [9, 10]. In 1982, Nusse and Varmus discovered the Wnt gene in mouse breast cancer cells, which was also known as the Int1 gene at the time [11]. Later studies found that the Int1 gene and the Drosophila Wingless gene (Wingless) were homologous genes, and finally the Int1 gene and the Wingless gene were collectively referred to as the Wnt gene [12]. Further studies revealed that Wnt proteins control a canonical signaling pathway through the key effector *ß*-catenin. Thus, the pathway is also known as the Wnt/*β*-catenin signaling pathway [13].

The Wnt signaling pathway includes the Wnt gene family and Wnt receptor. There are a total of 19 Wnt family members (Wnt1, Wnt2, Wnt3, Wnt4, Wnt5a, etc.) discovered so far, and these play critical roles in regulating proliferation, differentiation, growth, and so forth [14, 15]. Depending on its mode of action, Wnt signaling is classified as the canonical Wnt signaling pathway (the Wnt/*β*-catenin signaling pathway), the Wnt/Ca2+ signaling pathway, or the Wnt/PCP signaling pathway [16]. Among these, canonical Wnt/*β*-catenin signaling is the most important [17] and is the specific focus of this review.

The main members of Wnt/*β*-catenin signaling include Wnt protein, ubiquitin protein, *β*-catenin, frizzled (Fzd), casein kinase 1 (CK1), glycogen synthase kinase 3*β* (GSK-3*β*), axin, adenomatous polyposis coli (APC), disheveled (Dsh), T-cell factor/lymphocyte enhancer factor (TCF/LEF), and so on [18]. In the canonical Wnt signaling pathway, *β*-catenin is an important protein with transcriptional regulatory activity in the transduction pathway. The amount and phosphorylation state of *β*-catenin in the cell directly determines whether the canonical Wnt pathway is turned on or off [19].

As a homophilic adhesive complex to stabilize the cell contact surface, E-cadherin also plays a role in the Wnt signaling pathway [20]. Recent evidence indicates that the activity and expression levels of E-cadherin are critical in various cancers [21]. Cadherin is a calcium-dependent transmembrane glycoprotein that mediates the connections between epithelial cells [22]. E-cadherins can form dimers, and these zipper-like structures are the basis of cell adhesion. When the expression of E-cadherin is abnormal or the concentration of Ca2+ decreases, the dimers separate and the cell adhesion will decrease [23]. The mature E-cadherin structure includes a C-terminal intracellular domain, a transmembrane hydrophobic domain, and an N-terminal extracellular domain [24]. Its C-terminal intracellular domain forms a complex with multiple proteins, including *α*-catenin, pl20, actin, and *β*-catenin [25]. Therefore, E-cadherin can bind *β*-catenin, fix it on the cell membrane, and inhibit *β*-catenin from entering the nucleus, thereby antagonizing the Wnt signaling pathway, whereas loss of cadherin-mediated cell adhesion can promote *β*-catenin signaling [26, 27]. In vivo, the loss of E-cadherin can release *β*-catenin from its binding to the cell membrane [28, 29], which means that a reduction in the expression of E-cadherin can enhance nuclear *β*-catenin signaling events in the presence of Wnt. According to the model of canonical Wnt signaling, the accumulation of free cytoplasmic *β*‐catenin and its nuclear import are important steps. Within the nucleus, *β*-catenin specifically binds to proteins of the TCF/LEF family of transcription factors that activate the transcription of Wnt target genes [30]. Thus, the loss of E-cadherin can increase LEF/TCF-*β*-catenin signaling, which might be explained by cadherin and LEF/TCF having similar binding modes to *β*-catenin [31].

**Figure 1:**
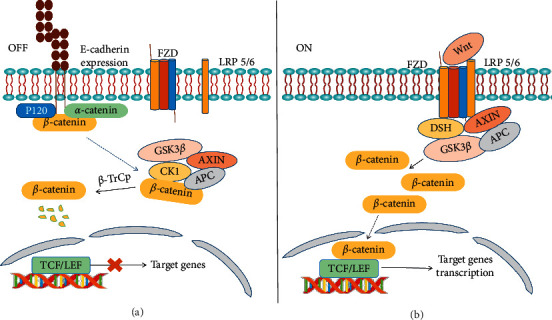
The Wnt/*β*-catenin signaling pathway. (a) “Wnt Signaling OFF.” Without Wnt activation, the destruction complex, composed of axin and its tumor suppressor partners APC, GSK-3*β*, and CK1, is formed. The destruction complex phosphorylates *β*-catenin and targets it for proteasomal degradation, thus maintaining low levels of *β*-catenin in the cytoplasm. In addition, the E-cadherin structure includes a *C*-terminal intracellular domain, a transmembrane hydrophobic domain, and an *N*-terminal extracellular domain. The *C*-terminal intracellular domain forms a complex with multiple proteins, including *α*-catenin, pl20, actin, and *β*-catenin. E-cadherin can bind *β*-catenin and fix it on the cell membrane, thus inhibiting *β*-catenin from entering the nucleus and antagonizing the Wnt signaling pathway. (b) “Wnt Signaling ON”. Wnt binds to Fzd/LRP5/6 receptors triggering the phosphorylation of Dsh, which is a negative regulator of the destruction complex. Dsh then recruits axin, which inhibits the formation of the destruction complex and allows *β*-catenin to accumulate in the cytoplasm and translocate into the nucleus where it binds to TCF/LEF and activates the transcription of Wnt target genes.

In normal mature cells, the Wnt pathway is turned off, and the destruction complex, which is composed of axin and its tumor suppressor partners APC, GSK-3*β*, and CK1, is formed. The destruction complex phosphorylates *β*-catenin and targets it for proteasomal degradation, thus maintaining low levels of cytoplasmic *β*-catenin. The graphical representation of these functions is shown in Figure 1(a). However, under pathological or other abnormal states, the Wnt signaling pathway can be triggered. Wnt proteins are secreted molecules that are acylated by porcupine and then bind to the seven-transmembrane receptor Fzd and lipoprotein-receptor-related protein (LRP) 5/6, thus activating the Dsh proteins. Dsh then recruits axin, which inhibits the formation of the destruction complex, thus allowing *β*-catenin to accumulate in the cytoplasm and translocate into the nucleus where it binds to TCF/LEF and activates the transcription of Wnt target genes like c-Myc, cyclin D1, MMP7, MMP9, and so forth, as shown in Figure 1(b). A considerable number of studies have shown that Wnt/*β*-catenin signaling is involved in controlling many cellular processes, including proliferation and differentiation, and thus is involved in the pathology of numerous diseases such as cardiac and vascular disease [32] and cancer to name just a few.

## 3. The Wnt/*β*-Catenin Signaling Pathway in OC

A large body of evidence suggests that compared with normal ovarian cells, Wnt pathway component proteins, such as Wnt ligand, Fzd, LRP5/6, and especially nuclear *β*-catenin protein, are significantly upregulated in OC [33]. The Wnt signaling pathway plays an important role in the embryonic development of ovarian tissue and in the proliferation, differentiation, and malignant transformation of ovarian cells [34]. The occurrence of OC is closely related to *β*-catenin in the Wnt pathway.

Existing studies have shown that *β*-catenin has dual functions. On the one hand, it can be used as a signal transduction molecule to mediate the transmission of Wnt signal from the cell membrane to the cytoplasm and nucleus [35, 36]. On the other hand, *β*-catenin also counteracts tumor formation, growth, invasion, and metastasis through its alternative function as a cytoskeletal component [37]. In normal somatic cells, *β*-catenin, with a rod-shaped supercoiled structure, forms a complex with E-cadherin at the cell membrane, which plays a role in maintaining the adhesion of homotypic cells and prevents cell movement [38]. Based on this, the Wnt/*β*-catenin pathway contributes to the occurrence and development of OC by upregulating the expression of *β*-catenin mRNA, whereas downregulating *β*-catenin reduces the proliferation activity of OC cells and prevents their migration and invasion.

The mechanism through which the Wnt/*β*-catenin pathway may be involved in regulating the occurrence and development of OC is mainly related to promoting proto-oncogene or cell regulatory factor gene transcription and mediating the expression of antiapoptotic genes [39]. Latifi et al. [40] found that the molecular structure of cells extracted from patients with metastatic OC was different from that of the primary tumor cells, showing the same gene characteristics as epithelial-mesenchymal transition (EMT), thus confirming that the Wnt/*β*-catenin pathway is one of the main signaling pathways involved in EMT. At the same time, the E-cadherin/*β*-catenin protein complex actively participates in the EMT and mesenchymal to epithelial transitions [41]. EMT is one of the basic mechanisms involved in organ fibrosis and cancer [42], and cell contact is a key determinant of EMT. The loss of E-cadherin promotes the release of *β*-catenin and thus promotes EMT, while the expression of E-cadherin can reverse the transformed phenotype [43–46], and thus the loss of cell-cell adhesion triggers EMT and is related to diseases involving EMT [41]. Studies have shown that inhibiting the expression of cell adhesion molecules (such as E-cadherin) and mesenchymal markers (such as vimentin) is a key process in EMT, while the positive EMT state (decreased E-cadherin expression) is a primary feature of OC and metastasis [47, 48].

Barghout et al. [49, 50] found that increasing the activity of *β*-catenin can induce carboplatin resistance in OC A2780 cells, while downregulating the expression of *β*-catenin prevents it from entering the nucleus, which effectively increases the sensitivity of OC cells to chemotherapeutic drugs. Decreasing *β*-catenin activity can also reverse the resistance of cancer cells to platinum-based chemotherapeutics. Therefore, downregulation of *β*-catenin, MMP7, survivin, cyclin, c-Myc, and other proteins in the Wnt/*β*-catenin signaling pathway can reverse EMT, inhibit the proliferation of OC cells, induce apoptosis, and reverse the effects of transformation therapy drug resistance. However, the underlying mechanisms for how *β*-catenin controls the development, proliferation, invasion, and metastasis of OC remain uncertain.

## 4. Plant-Derived Chinese Medicine Monomers on OC via Wnt/*β*-Catenin Signaling

Plant-derived Chinese medicine monomers play anticancer effects on regulating the Wnt/*β*-catenin signaling pathway, thereby inhibiting cell invasion, migration, autophagy, apoptosis, and cell cycle progression and promoting chemotherapy sensitivity and reversal of drug resistance. The roles of plant-derived Chinese medicine monomers on OC via the Wnt/*β*-catenin signaling are summarized in Table 1.

### 4.1. Resveratrol

Resveratrol is a phenolic substance isolated initially from *Veratrum grandiflorum* and is richly present in grapes, wine, peanuts, soy, and berries and has been attracting the attention of researchers for many decades [69]. Resveratrol has certain preventive and therapeutic effects against cancer through its antioxidation activity and by regulating metabolism [70, 71], and many studies have confirmed that resveratrol can inhibit the proliferation, invasion, and migration of OC cells and induce apoptosis. Wang and Shi [51] used the MTT method and flow cytometry to assess the effect of resveratrol on OC A2780 cells and found that the expression levels of *β*-catenin and c-Myc mRNAs and proteins were significantly reduced after treatment with 200 *μ*mol/L of resveratrol for 24 h. Hou et al. [52] treated OC SKOV3 cells with 20 *μ*mol/L, 40 *μ*mol/L, and 80 *μ*mol/L of resveratrol for 24 h and found that resveratrol could significantly inhibit the proliferation, invasion, and migration of OC SKOV3 cells as well as induce their apoptosis. At the same time, resveratrol also significantly reduced the mRNA expression levels of c-Myc, cyclin A, cyclin D1, N-cadherin, and vimentin and the protein expression level of *β*-catenin in cells, while the mRNA expression of p21, E-cadherin, and GSK-3*β* was significantly increased in a concentration-dependent manner. In addition, resveratrol could effectively inhibit the growth of OC CAOV3 and OVCAR3 cells and promote their apoptosis at a concentration of 120 *μ*M for 48 h. The expression level of *β*-catenin decreased significantly in both cell types, while the expression of Wnt2 protein was significantly decreased in CAOV3 cells but significantly increased in OVCAR3 cells. Taken together, these studies demonstrate that resveratrol can inhibit OC through Wnt/*β*-catenin signaling [71].

### 4.2. Hydroxysafflor Yellow A

Hydroxysafflor yellow A (HSYA) is among the major bioactive and water-soluble compounds isolated from Carthami flos, the flower of *Carthamus tinctorius* [72]. HSYA has various functions such as inducing tumor cell apoptosis, interfering with angiogenesis, and reversing drug resistance during transformation therapy [73, 74]. A rat model made by subcutaneously transplanting HO8910PM OC cells showed that 200 *μ*mol/L of HYSA for 24 hours inhibited cell growth and promoted apoptosis in HO8910PM cells [53]. At the same time, they showed that the expression of *β*-catenin, MMP7, and survivin were all downregulated and that the expression of the menin protein was upregulated in OC cells and in rat model tumor tissues. It is therefore suggested that HSYA inhibits the growth of OC cells and promotes their apoptosis through menin overexpression and inhibition of *β*-catenin expression, thus inhibiting the activation of the Wnt/*β*-catenin signaling pathway and reducing the downstream expression of the MMP7 and survivin proteins.

### 4.3. Emodin

Emodin is a natural anthraquinone derivative that occurs in many widely used Chinese medicinal herbs, such as *Rheum palmatum*, *Polygonum cuspidatum*, and *Polygonum multiflorum* [75]. It has various anticancer, antitumor, and anti-inflammatory effects protecting organs and tissues, and it is mostly used in basic cancer research or in combination with other anticancer therapies [76]. Hu [54] found that the proliferation of A2780 and SKOV3 cells treated with 20 *μ*M emodin was not significantly inhibited, but the invasion ability and EMT were significantly weakened. The epithelial indicators E-cadherin and keratin were significantly increased, the expression of the mesenchymal indicators vimentin, N-cadherin, MMP2, and MMP9 was significantly decreased, and the expression of p-GSK-3*β*, *β*-catenin, and ZEBI related to the EMT pathway was significantly decreased, suggesting that emodin inhibits EMT in epithelial OC cells by regulating the Wnt/*β*-catenin signaling pathway.

### 4.4. Oridonin

Oridonin (ORI) is an ent-kaurene tetracyclic diterpenoid compound isolated from *Rabdosia rubescens*, and it has various biological and pharmacological activities, including antitumor, antimicrobial, and anti-inflammatory effects [77]. In recent years, many *in vitro* experiments have shown that it has a significant inhibitory effect on more than 20 cancer cell lines [78, 79]. Liu and Guo [55] explored the effect of ORI on the migration and invasion of SKOV3 cells and found that ORI could significantly inhibit cell viability, induce apoptosis, and reduce cell migration. Their study also found that 5 *μ*mol/L, 10 *μ*mol/L, and 20 *μ*mol/L of ORI for 24 h increased the expression of E-cadherin and decreased the expression of vimentin, *β*-catenin, c-Myc, and cyclin D1 in a dose-dependent manner. This suggests that ORI might inhibit the Wnt/*β*-catenin signaling pathway and thereby inhibit the expression of related cytokines.

### 4.5. Schisandrin B

Schisandrin B is extracted from the Chinese medicine Schisandra, and it has pharmacological effects such as promoting tumor cell apoptosis, reducing inflammation and tissue edema, improving microcirculation and antioxidation, and expanding blood vessels [80, 81]. Zeng et al. [56] found that 10 *μ*mol/L, 20 *μ*mol/L, and 50 *μ*mol/L of schisandrin B for 48 h inhibited the proliferation of SKOV3 cells in both a time- and dose-dependent manner. Schisandrin B can also block cell cycle progression and reduce the protein expression levels of *β*-catenin, c-Myc, and cyclin D1. These results suggest that schisandrin B may reduce the proliferation of SKOV3 cells and block cell cycle progression by inhibiting the Wnt/*β*-catenin signaling pathway.

### 4.6. Apigenin

Apigenin is a flavonoid derived from vegetables, fruits, tea, and beans [82], and it has some effect on preventing and treating cancer, reducing the toxicity of chemotherapy, and reversing drug resistance [83]. Zhang et al. [57] found that 30 *μ*mol/L of apigenin for 24 h could effectively inhibit the migration and invasiveness of HO8910 OC cells, and it can also downregulate *β*-catenin and E-cadherin, which are the downstream effectors of the Wnt signaling pathway. The expression level modulation of genes and proteins may therefore be achieved by inhibiting the Wnt/*β*-catenin signaling pathway. Indeed, cytological analysis, western blotting, and immunofluorescent staining all suggest that apigenin induces autophagy-mediated downregulation of *β*-catenin in treated cells, thereby inhibiting the Wnt/*β*-catenin signaling pathway [58].

### 4.7. Tea (*Camellia sinensis*) Flower Saponins

Tea (*Camellia sinensis*) flower saponins (TFS) has antiallergic and antitumor effects [84, 85]. Chen et al. [59, 60] studied the effects and mechanisms of TFS on the proliferation and differentiation of ovarian cancer stem-like cells (OCSLCs) and found that doses of 2.5 *μ*g/ml, 3.0 *μ*g/ml, 3.5 *μ*g/ml, and 4.0 *μ*g/ml reduced the viability of OCSLCs compared with the control group. TFS inhibited clonal expansion, and tumor sphere formation reduced the cells' self-renewal capacity and was shown to downregulate the expression of p-AKT, p-GSK-3*β*, *β*-catenin, and c-Myc proteins while upregulating the phosphorylation of *β*-catenin thereby inhibiting the Wnt/*β*-catenin signaling pathway. It is therefore suggested that TFS can inhibit the growth and proliferation of OCSLCs and reduce their stem-like characteristics through inhibition of the Wnt/*β*-catenin signaling pathway.

### 4.8. Icariin

Icariin is the principal active ingredient in the Chinese medicine Epimedium. As a new type of flavonoid anticancer drug, it has demonstrated significant antitumor effects [86]. Chen et al. [61] found that 20 *μ*g/ml, 40 *μ*g/ml, and 60 *μ*g/ml of icariin for 24 h could significantly inhibit the growth and proliferation of CAOV3 OC cells, while RT-PCR showed that icariin could reduce *β*-catenin mRNA transcription (thus inhibiting the transcription of the Wnt signaling pathway target genes c-Myc and cyclin D1) and Western blot confirmed that the compound could downregulate the protein expression of *β*-catenin, c-Myc, and cyclin D1. These results suggest that icariin can inhibit the proliferation of human CAOV3 cells and that this might be achieved by inhibiting the Wnt/*β*-catenin signaling pathway.

### 4.9. Epigallocatechin-3-gallate

Epigallocatechin-3-gallate (EGCG) is extracted from green tea and has been shown to have multiple effects on both pathological and physiological processes in humans [87]. In recent years, a large number of studies have confirmed that EGCG has a strong pharmacological effect on the prevention and treatment of tumors [88]. Long and Tang [62] studied the effects of EGCG on the proliferation of HO8910 OC cells and Wnt/*β*-catenin signaling pathway-related gene expression in the cells. They found that EGCG had a strong antiproliferative effect and that 40 *μ*g/ml of EGCG the cell cycle of HO8910 cells was completely blocked. At the same time, EGCG could significantly reduce the level of *β*-catenin and cyclin D1 mRNA and protein. These results suggest that the mechanism through which EGCG inhibits the growth of HO8910 OC cells may be related to the inhibition of the Wnt/*β*-catenin signaling pathway.

### 4.10. Paeonol

Paeonol (PAE) is one of the active components of Cortex Moutan, which has various anti-inflammatory, antioxidant, and antitumor effects [89]. Studies have confirmed that 0.4 mmol/L, 0.8 mmol/L, and 1.6 mmol/L of PAE for 24 h, 48 h, and 72 h can inhibit the proliferation of A2780 OC cells and promote their apoptosis in a time- and dose-dependent manner. PAE also has an effect on the occurrence and development of OC. Related studies have found that a certain concentration of PAE can inhibit the proliferation of human A2780 OC cells and induce their apoptosis, block the cells in S phase, and significantly reduce the expression of *β*-catenin and c-Myc proteins. The results of a scratch test showed that the migration ability of the A2780 cells decreased significantly proportionally to the drug concentration and exposure time. These results confirmed that PAE inhibits the Wnt/*β*-catenin signaling pathway by regulating the expression of related proteins, thereby inhibiting the growth of A2780 cells and inducing apoptosis [63, 64].

### 4.11. Tetrandrine

Tetrandrine (TET) is a Chinese medicine isolated from the root of *Stephania tetrandra* S. Moore, [90]. Modern pharmacological studies have found that TET acts as a calcium channel blocker and has immunosuppressive, anti-inflammatory, antioxidative, and anticancer activities [91]. Wang et al. [65] found that when A2780 OC cells and ES-2 ovarian clear cancer cells were cultured for 48 h, their survival rates decreased significantly with increasing TET concentration. For the A2780 cells, 5.0 *μ*mol/L of TET inhibited the migration and invasion ability and decreased the levels of MiR-21, p-GSK3*β*, *β*-catenin, N-cadherin, and vimentin, whereas for ES-2 ovarian clear cancer cells, 3.0 *μ*mol/L TET could do the same. Meanwhile, the E-cadherin protein expression level was significantly increased. Similarly, Jiang and Hou [66] also found that TET could enhance the sensitivity of SKOV3/PTX cells to PTX by inhibiting the *β*-catenin/c-Myc/cyclin D1 signaling pathway.

### 4.12. Proanthocyanidins

Proanthocyanidins (PCs) are commonly found in plants such as metaplasia, grape seeds, pine bark, and sorghum bark [92]. PCs are internationally recognized natural antioxidants with high bioavailability and low toxicity, and they have been shown to protect against free radical-related diseases [93, 94]. Recent studies have shown that PCs can inhibit the growth of a variety of tumor cells, promote tumor cell apoptosis, and also antagonize the toxicity of chemotherapeutics in healthy cells achieving good anticancer effects with low toxicity [95]. Zhang et al. [67] found that a 24 h treatment with 10 *μ*g/ml of PC extracted from the bayberry leaf could inhibit *β*-catenin, cyclin D1, and c-Myc protein expression, thereby impeding the self-renewal ability of drug-resistant OVCAR3 OC cells, weakening their stem cell characteristics, blocking the cell cycle, and reversing drug resistance. The suggested mechanism for these effects is related to inhibition of the Wnt/*β*-catenin signaling pathway.

### 4.13. Naringin

Naringin is a natural flavonoid that mainly exists in the peel and pulp of grapefruit and lime and has exhibited antioxidative, anti-inflammatory, and antitumor effects [96, 97]. Naringin can inhibit the proliferation of a variety of tumors, such as cervical cancer [98] and OC [99], and was shown to inhibit the proliferation of cisplatin resistant OC cells (SKOV3/CDDP) in a dose- and time-dependent manner. When combined with cisplatin at 20 mol/L, naringin could reduce the expression of Cyclin D1, c-Myc, and *β*-catenin in SKOV3/CDDP cells and partially reverse cisplatin resistance. The mechanism of this activity may be related to the Wnt/*β*-catenin signaling pathway. In addition, the combination of naringin with cisplatin might prevent cell cycle progression, thereby inhibiting the proliferation, invasion, and migration of OC cells [68].

## 5. Conclusions and Future Perspectives

The Wnt/*β*-catenin pathway is an important target for treating OC, and many studies have investigated the potential therapeutic effects of antibodies and small molecules that target this pathway, with some currently being tested in clinical trials [100]. In recent years, accumulating studies have concentrated on the effect of plant-derived Chinese monomers in treating OC because of their limited side effects and better clinical efficacy. The current evidence suggests that plant-derived Chinese monomers can act on different molecular targets within the Wnt/*β*-catenin signaling pathway to inhibit the metastasis and proliferation of OC.

Plant-derived Chinese monomers play a role in the development of new targeted therapies for the prevention and treatment of OC, but there are still some limitations to these studies. Although some mechanisms of plant-derived Chinese monomers in antitumor therapy have been discussed, the research on plant-derived Chinese monomers' anticancer effects and their ability to reverse transformation therapy resistance are mostly based on experiments involving *in vitro* cultured cells. Regarding the cytotoxicity of plant-derived Chinese monomers, their bioavailability is not very clear, and further *in vitro*, *in vivo*, and clinical studies are needed. The anticancer effect of plant-derived Chinese monomers is multitargeted. At present, the most common problems in research on the antitumor effects of plant-derived Chinese monomers are insufficient validation of findings and the scarcity of clinical trials. Therefore, the anticancer effects of these monomers should be further demonstrated at different research levels. Carrying out clinical trials for plant-derived Chinese monomers with definite curative effects that are both stable and obvious will promote the translation of experimental research results into clinical practice and lay a solid modern medical theory foundation for the clinical application of plant-derived Chinese monomers against cancer. Thus, the specific mechanisms through which plant-derived Chinese monomers influence the Wnt/*β*-catenin signaling pathway require deepened studies in the future.

## Figures and Tables

**Table 1:** Mechanism of plant-derived Chinese medicine monomers on OC via the Wnt/*β*-catenin signaling pathway.

Plant-derived Chinese medicines	Targets	Mechanism	Refs.
Resveratrol	c-Myc, cyclin A, cyclin D1, N-cadherin, vimentin, *β*-catenin, E-cadherin, p21, Wnt3a, GSK-3*β*	Inhibiting proliferation, invasion, and migration; inducing apoptosis	[51, 52]
Hydroxysafflor yellow A	*β*-Catenin, MMP7, survivin, menin	Inhibiting proliferation; inducing apoptosis	[53]
Emodin	E-cadherin, keratin, vimentin, N-cadherin, MMP2, MMP9, p-GSK-3*β*, *β*-catenin, ZEBI	Inhibiting invasion; reversal of EMT	[54]
Oridonin	Vimentin, c-Myc, cyclin D1, E-cadherin, *β*-catenin	Inhibiting proliferation, invasion and migration; inducing apoptosis	[55]
Schisandrin B	*β*-Catenin, c-Myc, cyclin D1	Inducing apoptosis; blocking cell cycle progression	[56]
Apigenin	*β*-Catenin, E-cadherin	Inhibiting invasion and migration	[57, 58]
Camellia sinensis	p-AKT, p-GSK-3*β*, *β*-catenin, c-Myc, p-*β*-catenin	Inhibiting proliferation	[59, 60]
Icariin	*β*-Catenin, c-Myc, cyclin D1	Inhibiting proliferation	[61]
Epigallocatechin-3-gallate	*β*-Catenin, cyclin D1	Inducing apoptosis; blocking cell cycle progression	[62]
Paeonol	Bcl-2, Bax, *β*-catenin, c-Myc	Inhibiting proliferation and migration; inducing apoptosis; blocking cell cycle progression	[63, 64]
Tetrandrine	p-GSK3*β*, *β*-catenin, E-cadherin, N-cadherin, vimentin	Inhibiting proliferation, invasion, and migration; reversal of EMT	[65, 66]
Proanthocyanidins	*β*-Catenin, cyclin D1, c-Myc	Inhibiting proliferation; inducing apoptosis; blocking cell cycle progression; reversing transformation therapy resistance	[67]
Naringin	*β*-Catenin, cyclin D1, c-Myc	Inhibiting proliferation, invasion, and migration; reversing transformation therapy resistance	[68]

## Data Availability

No data were used to support this study.
